# Effect of a Personalized Mobile Health Intervention Using Artificial Intelligence (the WARIFA App) Versus a Nonpersonalized Intervention on User-Defined Objectives, Healthy Lifestyles, and Management of Type 1 Diabetes (T1D): Protocol for a Randomized Controlled Trial

**DOI:** 10.2196/84510

**Published:** 2026-05-22

**Authors:** Carmelo Betancort Acosta, Garlene Zamora Zamorano, María Luisa Álvarez Malé, Lilisbeth Perestelo-Perez, Alezandra Torres Castaño, Marit B Veierød, Kristina Reyes Suárez, Alejandro Déniz García, Eirik Årsand, Inger Torhild Gram, Maja-Lisa Løchen, Cristina Soguero-Ruiz, André Henriksen, Antonio J Rodríguez Almeida, Miroslav Muzny, Reetta Välimäki, Thomas Schopf, Himar Fabelo, Conceição Granja, Ana M Wägner

**Affiliations:** 1 Department of Endocrinology and Nutrition Complejo Hospitalario Universitario Insular-Materno Infantil Las Palmas de Gran Canaria, Las Palmas Spain; 2 The University Institute of Biomedical and Healthcare Research Universidad de Las Palmas de Gran Canaria Las Palmas de Gran Canaria, Canary Islands Spain; 3 Instituto de Investigación Sanitaria de Canarias Las Palmas de Gran Canaria Spain; 4 Evaluation Unit Canary Islands Health Service Santa Cruz de Tenerife Spain; 5 Network for Research on Chronicity, Primary Care, and Health Promotion Santa Cruz de Tenerife Spain; 6 Fundación Canaria Instituto de Investigación Sanitaria de Canarias Las Palmas de Gran Canaria Spain; 7 Oslo Centre for Biostatistics and Epidemiology Department of Biostatistics, Institute of Basic Medical Sciences University of Oslo Oslo Norway; 8 Department of Computer Science UiT The Arctic University of Norway Tromsø Norway; 9 Norwegian Centre for E-health Research Tromsø Norway; 10 Department of Community Medicine UiT The Arctic University of Norway Tromsø Norway; 11 Department of Clinical Medicine UiT The Arctic University of Norway Tromsø Norway; 12 Department of Cardiology University Hospital of North Norway Tromsø Norway; 13 Department of Signal Theory and Communications Telematics and Computing Systems Rey Juan Carlos University Madrid Spain; 14 Research Institute for Applied Microelectronics Universidad de Las Palmas de Gran Canaria Las Palmas de Gran Canaria Spain; 15 Sensotrend Oy Tampere Finland; 16 Research Unit Hospital Universitario de Gran Canaria Doctor Negrín Las Palmas de Gran Canaria Spain; 17 Faculty of Nursing and Health Sciences Nord University Bodø Norway

**Keywords:** noncommunicable diseases, type 1 diabetes, mHealth app, mobile health, artificial intelligence, behavior change, lifestyle, user-centered

## Abstract

**Background:**

Noncommunicable diseases are the leading cause of death worldwide. Cardiovascular and respiratory diseases, cancer, and type 2 diabetes share common risk factors that can be addressed: physical activity, a healthy diet, and avoiding smoking and alcohol. The Watching the Risk Factors (WARIFA) mobile health app was created for general health awareness and to support users in adopting healthier behaviors, as well as to support type 1 diabetes (T1D) self-management.

**Objective:**

This study aims to evaluate the effectiveness of the WARIFA app with personalized artificial intelligence (AI)–driven messages, compared to a nonpersonalized version, in promoting health-related behavior change among the general population and individuals with T1D.

**Methods:**

A total of 108 European participants, including individuals with T1D, were to be randomized (computer-generated sequence, double-blind, 1:1 ratio) to an intervention or control group. In the intervention group, participants used the WARIFA app with personalized messages and the use of AI. This applied to certain functionalities, such as providing recommendations on healthy dietary habits based on food logging and offering advice and encouragement through daily step tracking. It also provided risk predictions for cardiovascular and respiratory diseases, cancer, and type 2 diabetes. Participants with T1D were offered glucose predictions based on previous measurements. In the control group, participants used a WARIFA app without personalized messages or AI. Both WARIFA app versions offered access to air quality and UV index information for the geographical area, as well as displaying physical activity in the form of daily steps and sleep hours, as well as glucose results for participants with T1D. Both groups were provided with an activity monitor and used the WARIFA app for 8-12 weeks. The primary outcome is a self-defined goal, chosen from a set of proposed objectives at baseline and assessed at the end of the study using a Likert scale (1 to 10 points, 0 being no achievement at all and 10 being full achievement of the objective). Secondary outcomes include: engagement with the app, changes in lifestyle behavior, body composition, lipid profile, glycated hemoglobin (T1D only), hypoglycemic events (T1D only), and health-related quality of life, as well as acquired knowledge, self-efficacy, and usability.

**Results:**

The clinical trial took place between January and June 2025. A total of 88 participants were finally recruited. The data are being analyzed, and the results are expected to be published in 2026.

**Conclusions:**

There is evidence that improving lifestyle behavior can prevent noncommunicable diseases. In this study, we aim to evaluate the effectiveness of the WARIFA app to improve lifestyle behaviors and T1D management.

**Trial Registration:**

ClinicalTrials.gov NCT06918444; https://clinicaltrials.gov/study/NCT06918444

**International Registered Report Identifier (IRRID):**

DERR1-10.2196/84510

## Introduction

### Background

Chronic noncommunicable diseases (NCDs) are a major global health problem. They are the leading cause of mortality and morbidity, accounting for 74% of all deaths worldwide [1]. Their management and treatment are a major economic concern, accounting for 25% of the total health care budget in Europe [2]. The major NCDs are cardiovascular diseases (CVDs), respiratory diseases, cancer, and type 2 diabetes (T2D).

CVD is the leading cause of death worldwide and in Europe, accounting for 32.7% of all deaths in 2022 [3]. Ischemic heart disease and cerebrovascular disease are the most common causes, accounting for 32.4% and 20.8% of CVD deaths, respectively [3]. There is variability between high-income and middle-income countries in the proportion of premature CVD deaths, with the proportion being higher in middle-income countries in both sexes (36% in women and men in middle-income countries, vs 16% and 24% in women and men in high-income countries, respectively) [4]. In 2019, these diseases were responsible for an estimated 70 million disability-adjusted life years in European Society of Cardiology member countries [4]. There is also an unequal distribution of CVD deaths in Europe, with more than half of all deaths occurring in eastern countries, compared with 23% in northern and western countries [1,4,5]. Metabolic risk factors include hypertension, hyperglycemia, hyperlipidemia, and obesity. Lifestyle risk factors include tobacco and alcohol use, physical inactivity, and an unhealthy diet.

Respiratory diseases are the third leading cause of death in Europe, accounting for 7% of all causes of death in 2022 [3]. Chronic obstructive pulmonary disease (COPD) and asthma are the most common respiratory diseases. COPD reduces quality of life and increases morbidity, hospital admissions, and premature death. Most COPD is preventable, and the main risk factors are smoking and air pollution [3,6].

Despite a 11.9% decrease in the standardized mortality rates between 2012 and 2022, cancer is still the second most frequent cause of death in the European Union (EU), accounting for 22.3% of all deaths in 2022. Lung, colorectal, and breast cancer account for 19.8%, 11.5%, and 7.4% of all deaths from cancer in the EU [3]. Globally, the most commonly diagnosed group of cancers is skin cancer, and cutaneous melanoma, the deadliest, accounts for 20% of skin cancers [7]. As with CVD, many cancers can be prevented by adopting a healthy lifestyle. Smoking, alcohol, physical inactivity, and an unhealthy diet, the latter 2 leading to overweight and obesity, are responsible for the majority of cancer incidence [4]. In addition, UV radiation exposure is the leading environmental cause of melanoma, a type of cancer that continues to increase in fair-skinned populations worldwide [7].

T2D has a prevalence of 5%-20% in the adult population. It is associated with other NCDs and includes risk factors such as smoking, physical inactivity, unhealthy diet, and obesity. Prevention can reduce the risk of diabetes by up to 78% and reduce cardiovascular mortality, cancer mortality, and the incidence of CVD in people with T2D [8,9].

For type 1 diabetes (T1D), there are no established recommendations for prevention. However, controlling risk factors such as smoking, alcohol consumption, sedentary lifestyle, and unhealthy diet improves disease control and delays the onset of complications such as CVD [10-13]. Therefore, while modifying these lifestyle factors does not prevent the onset of T1D, it does reduce the risk of NCDs in this specific population. It improves glycemic control and prevents other microvascular complications that are more prevalent in people with T1D, such as diabetic retinopathy [14-16]. Treatment of T1D requires a high degree of self-care, which can represent a burden and affect health-related quality of life [17]. The incorporation of technology such as continuous glucose monitoring (CGM) and automated insulin delivery systems facilitates diabetes management by providing relevant information and improved glycemic control [18,19].

The risk of developing some NCDs can be assessed based on known risk factors. This is important as we can act on those risk factors that are modifiable, provide personalized recommendations for each individual, and prevent or postpone the onset of NCDs, reducing morbidity and mortality and economic expenditure. Furthermore, the 4 major NCDs share common risk factors: physical inactivity, an unhealthy diet, and alcohol and tobacco consumption. Addressing these 4 risk factors could reduce the risk of NCDs.

An increasing number of people use mobile apps to improve their health. These apps offer direct access to information on specific health topics, as well as useful features, such as medication reminders, physical activity, and vital sign recording [20]. Artificial intelligence (AI) is also becoming an increasingly popular tool. Some studies have examined the potential application of AI in mobile health apps, particularly in relation to mental and oral health [21].

Watching the Risk Factors (WARIFA) [22] is a project funded by the European Commission and coordinated by the Norwegian Centre for E-Health Research. It involves 12 partners from 6 European countries. It aims to provide early risk assessment to enable individualized risk prevention. A mobile health (mHealth) app (the WARIFA app) has been developed, which uses user-generated data analysis and AI to provide NCD risk estimation, as well as personalized messages, based on scientific evidence, to promote healthy behaviors.

Personalized recommendations in health apps are highly relevant as they allow feedback to be tailored to each user’s actual needs in terms of content, intensity, and timing. This can lead to greater engagement, self-efficacy, and adherence to recommended behaviors. Unlike static or nonpersonalized recommendations, just-in-time, adaptive interventions provide the right recommendations at the right time, taking the context of each user into account. This type of intervention is commonly used in health apps [23-26]. Thus, personalized interventions provide more than just general information; they also seek to maximize the timeliness and relevance of each recommendation by finding the most appropriate moment to encourage adherence. The WARIFA app takes this approach by integrating automatically received data (from activity devices) and user-entered data to continuously adapt its recommendations. For example, it adjusts physical activity suggestions according to daily steps and generates dietary recommendations according to daily consumption records.

Co-creation methodology was used during the development of the WARIFA app. This involved the users throughout the process, enabling their needs to be identified and feedback to be obtained. Eighteen focus groups consisting of 3-10 participants of different ages from the general population and people with T1D were organized for this purpose. The co-creation process was divided into 2 phases.

In the first phase, focus groups were held with participants who expressed their needs and expectations for a health app and created hand-drawn sketches and prototypes. In the second phase, focus groups were held with a prototype of the app based on users’ preferences from the previous phase. Quantitative and qualitative information obtained from each group was used to update the app, creating one that aligns with the real needs of the population.

After the co-creation process, the randomized controlled trial (RCT) whose protocol is described in this article was conducted to evaluate the effectiveness of the WARIFA app in promoting healthy lifestyles and in the self-management of T1D.

### Objective and Hypothesis

The aim of this RCT is to evaluate the effectiveness of the WARIFA app in promoting healthy lifestyles and in the self-management of T1D.

The hypothesis is that use of the WARIFA app will improve healthy eating habits, increase physical activity, reduce alcohol consumption, promote smoking cessation, and improve sun protection behaviors. In addition, it is expected to reduce hypoglycemic events and lower glycosylated hemoglobin A_1c_ (HbA_1c_) in people with T1D.

## Methods

### Overview

This study protocol was developed in accordance with the recommendations for interventional trials 2013 statement SPIRIT (Standard Protocol Items: Recommendations for Intervention Trials) [27] and SPIRIT-AI (SPIRIT–Artificial Intelligence) guidance published for trials of AI interventions [28].

### Study Design and Setting

A randomized, controlled, double blind, parallel, multicenter trial was conducted in 2 populations (general population and individuals with T1D) in 3 European countries. For this purpose, participants were provided either with the WARIFA app with personalized recommendations or the same WARIFA app without personalized recommendations. The participating centers are: the Research Institute for Biomedical and Health Sciences, University of Las Palmas de Gran Canaria, Gran Canaria, Spain, associated with Complejo Hospitalario Universitario Insular Materno-Infantil; and the National Center for eHealth, Tromsø, Norway. In Romania, it is led by Netsun.

### Participants and Recruitment

Participant eligibility criteria for recruitment are presented in Textbox 1.

**Inclusion and exclusion criteria.**

**Inclusion criteria**

- Common to both populations: adults aged 18 years and older with an Android smartphone and internet access.
- General population group: healthy or diagnosed with no more than one noncommunicable disease (NCD; cardiovascular disease [CVD], cancer, type 2 diabetes [T2D], or chronic obstructive pulmonary disease [COPD]).
- Type 1 diabetes (T1D) group: diagnosed with T1D, treated with multiple insulin injections, and using a continuous glucose monitor of the brand Freestyle Libre (FSL; Abbott).

**Exclusion criteria**

- Common to both groups: individuals aged 18 years and younger, pregnancy, mobile phone incompatible with the WARIFA (Watching the Risk Factors) app (eg, Apple devices), not understanding any of the languages used in the app (English, Spanish, Norwegian, and Romanian), or any other reason that precludes follow-up.
- General population: people with 2 or more NCDs (CVD, cancer, T2D, or COPD).

### Randomization and Blinding

After registration in the WARIFA app, participants were randomly assigned in a 1:1 ratio by the system, using a computer-generated randomization list. This randomization was stratified by T1D status (yes or no) and center, to ensure balance within each group. The app was downloaded from Google Play, with the same download link for all participants, who were unaware of what exactly to expect from the app. Once downloaded, and after registration, eligibility criteria were confirmed, informed consent was given within the app and participants were randomized: one feature set in the app was available for the control group and another for the intervention group. This procedure was done individually by the participants, avoiding interaction amongst them. Both the researchers and the participants were unaware of group allocation, and the researchers who analyze the data are blinded, too.

In the Norwegian and Romanian centers, the app was downloaded either independently by the participant or with assistance (Norway). In the Spanish center, visits were face-to-face, as blood tests and physical examinations were carried out.

### Intervention

The intervention consists of 12 weeks using a version of the WARIFA app with personalized risk estimations for different NCDs and individualized recommendations, based on each participant’s personal preferences.

To do this, the app uses a simple and intuitive interface, using data available from public databases (for UV index and air quality index) and data from the participants themselves (entered manually, via questionnaires, or automatically, via sensors). The app uses AI to create rule-based recommendations and feedback based on scientific evidence and behavior change models. Bayesian belief networks are used to assess individual risk for CVD in participants with T1D [29], and deep learning for the prediction of glucose values based on previous CGM data [30].

The WARIFA app is downloaded to participants’ own smartphones, who are also provided with a physical activity monitor (Samsung Galaxy Fit 3) to track physical activity, heart rate, and sleep. This is connected to the WARIFA app. Participants with T1D use their glucose sensors (Free Style Libre [FSL] 2 and 3, Abbott), also linked to the WARIFA app.

Participants aimed to use the app for 12 weeks, with 2 face-to-face visits (in Spain only): one at the start of the study and one at the end of the study. At the first visit, the app was downloaded and linked to the activity monitor (all participants) and glucose monitors (T1D participants only). Both visits included blood tests and a physical examination. The Norwegian and Romanian centers did not perform in-person studies, and all participant information was provided through the app in these countries. Questionnaires were completed within the app itself in all centers.

**Figure 1:**
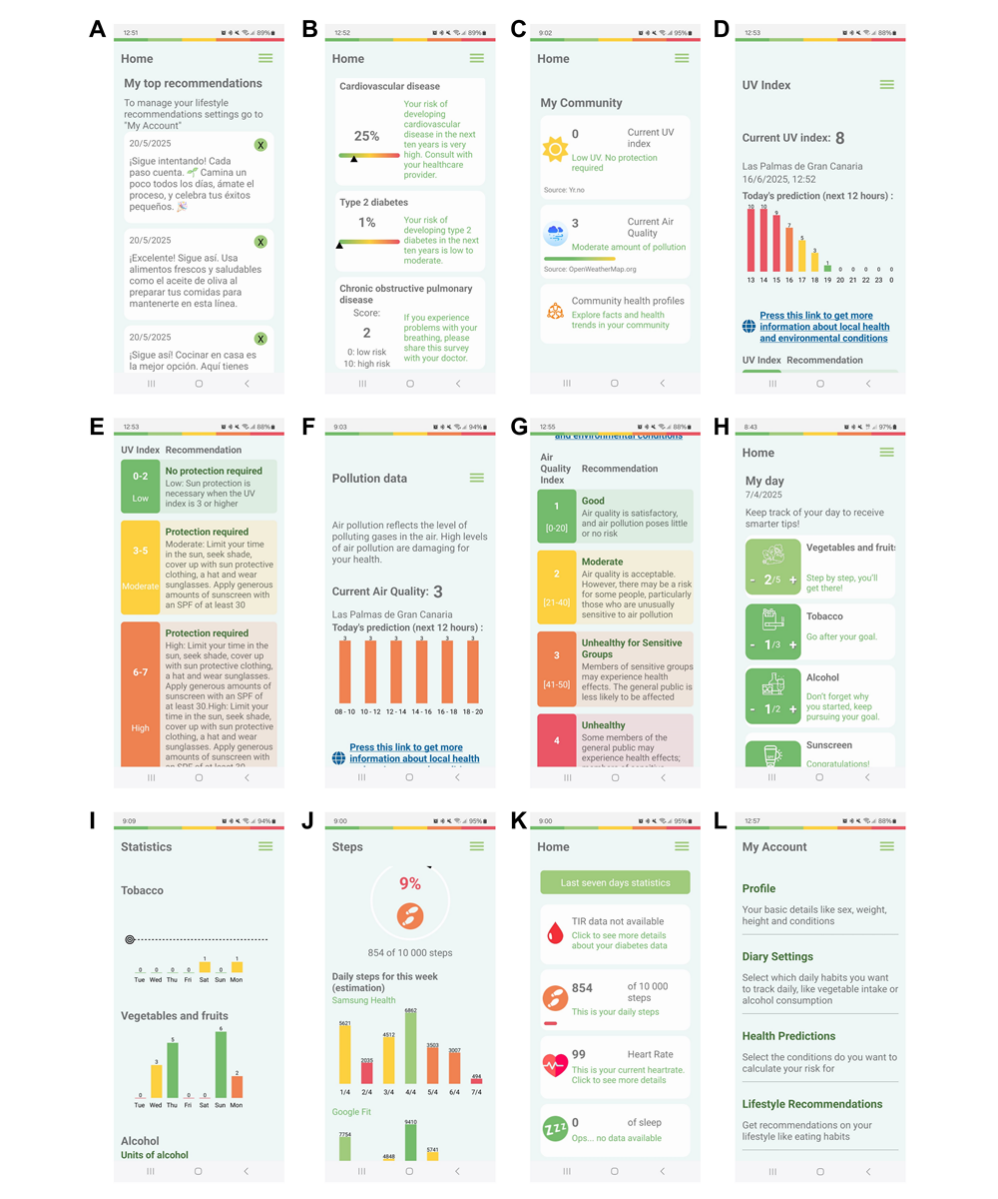
Functions of the WARIFA app: (A) Personalized recommendations; (B) Risk calculators for different noncommunicable diseases and chronic obstructive pulmonary disease screening; (C) My community functionality, including the UV index (D) and recommendations (E); and (F) air quality with recommendations (G). (H) Diary functionality and viewing statistics for the last 7 days (I). (J) Steps functionality and comparison with the previous seven days. (K) The main. (L) Options menu.

**Figure 2:**
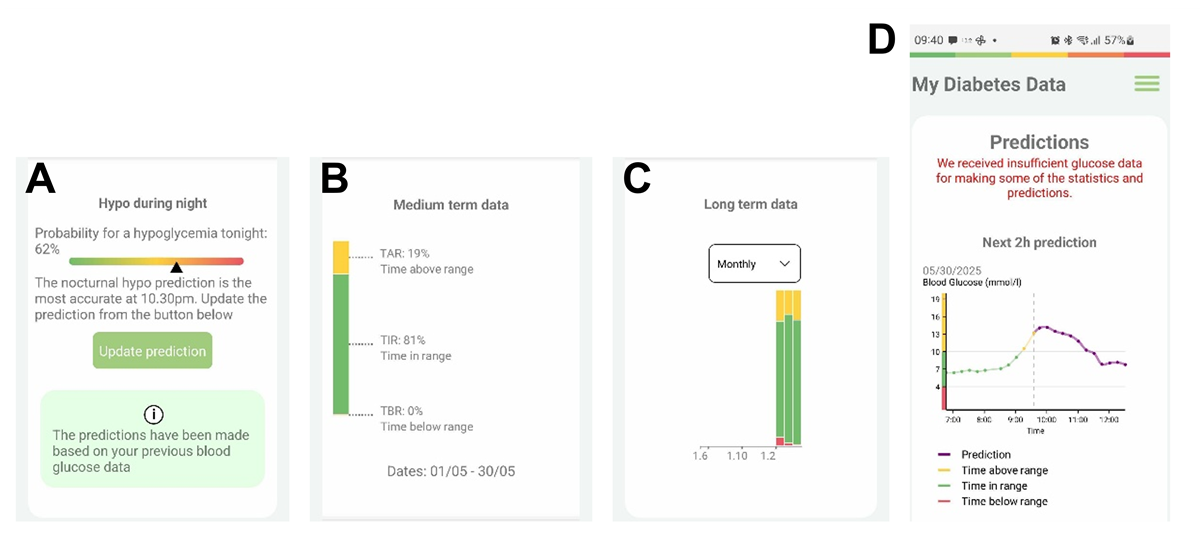
Diabetes features: (A) Overnight hypoglycaemia prediction functionality; (B) display of % range times in the last month; (C) comparison of monthly range times; and (D) glucose prediction functionality.

The WARIFA app includes several functionalities (Figure 1):

1. My day: a diary is available to record various items [Figure 1H]. Participants are able to easily and intuitively record their fruit and vegetable intake, alcohol and tobacco consumption, and sunscreen use every day. They are able to customize the diary to choose the items they want to record. They see a graphical presentation of the records of the last 7 days, with a marker for their personal goal [Figure 1I], as well as individual and personalized messages generated with the use of AI for the improvement of their goals. People with T1D can also record their hypoglycemic events.
2. Assess your lifestyle: participants complete questionnaires that provide data for WARIFA to personalize their risk estimation and recommendations. These questionnaires include basic variables (age, gender, weight, and height), lifestyle habits (alcohol consumption, smoking, and physical activity), eating habits, and sun exposure, as well as additional risk factors for melanoma. Once the questionnaires have been completed, the app delivers the first messages with personalized recommendations. If the user enters this section at another stage in the trial and changes data in the questionnaires, new recommendations are generated and delivered to the user.
3. My top recommendations: personalized recommendations appear for each participant each day, based on the data obtained through the initial questionnaires and how far they are from the recommended target for each item (Figure 1A). These recommendations are based on scientific evidence and updated World Health Organization guidelines.
4. My health predictions: will give access to risk calculators (Figure 1B) for different NCDs (T2D, CVD, COPD, and melanoma) with personalized risk estimation over the next 10 years. During the WARIFA project, some risk calculators were developed, including the cardiovascular risk calculator for T1D [29]. The melanoma risk calculator was developed based on the first primary risk prediction tool created by the Melanoma Institute Australia [31]. The FINDRISK calculator, which has been validated in several European countries, is used to calculate the risk of T2D [32]. To calculate cardiovascular risk in the general population, the SCORE2/SCORE2OP calculator was used; this has been validated in the European countries participating in the study [33,34]. Finally, the self-scored COPD population screener questionnaire [35] was used to screen for COPD in smokers, since there is no COPD risk calculator validated in the European population. In all cases, the personalized risk of the different NCDs is accompanied by an explanatory message. If the COPD-Q score is high, it is recommended that participants visit their health service physician for further assessment.
5. My community: through the use of geolocation (if permission is granted), it provides personalized recommendations based on the UV and air quality indices in the participant’s geographical area (Figures 1C,1D,1E,1F, and 1G). A link to a web page [36] with additional health-relevant information (air pollution and UV rays, statistics on NCDs of the population, lifestyle habits...) about the area where the user is located is also displayed.
6. Step functionality: shows the number of steps taken by participants at any point in each day, as well as a graphical presentation of the number of steps for the last 7 days (Figure 1J). Recording is automatic via the physical activity monitor.
7. Heart rate function: shows the current heart rate and a graph with its evolution in relation to the measurements in the last 24 hours. The source of the data is the physical activity monitor.
8. Sleep function: shows today’s hours of sleep and a bar graph of the hours of sleep the last 7 days recorded by the physical activity monitor.
9. Diabetes functionality: people with T1D can view their glucose data in near real time (with a delay of 30 minutes) by connecting to the glucose sensor (Figure 2). They can also view historical glucose data through various graphs, such as time in different ranges (Figures 2B and 2C), glucose management index, and hypoglycemic events. Using AI, they receive an estimate of the interstitial glucose prediction over the coming 30 minutes and 2 hours (Figure 2D), as well as a personalized risk of nocturnal hypoglycemia (Figure 2A).
10. Settings and other functions: in this section, participants can customize which parts of the WARIFA app they want to see, as well as the notifications, sounds, fonts, and language used.

### Control Group

The control group received a low-intensity intervention, with access to a WARIFA app version where almost all the functionalities mentioned above are available, except for the diary, risk calculators, and personalized recommendations. AI features such as near real-time glucose prediction, night time hypoglycemia risk, and motivational feedback in the top recommendations section are not available in this version, either. Data are collected in a similar manner, and an activity monitor is provided for the duration of the study, too.

### Patient and Public Involvement

Potential end-users, including a T1D patient association, were involved during the co-creation of the WARIFA app. The results of the RCT will be presented in an open access research article, as well as on the project website and on the European Commission’s CORDIS platform [37].

### Outcomes

#### Overview

The primary outcome is a participant-defined goal, whose fulfillment is assessed at the end of the study using a Likert scale ranging from 1 (“far below expectations”) to 10 (“far above expectations”) [38].

Participants can choose their own goal from a range of options: increase fruit and vegetable consumption, increase physical activity, reduce alcohol consumption, stop smoking, improve sun protection behavior, and reduce the number of weekly hypoglycemic episodes (the latter only for T1D participants).

The secondary outcomes are: frequency of app use, time spent using the app, consistency in data recording, changes in eating habits, increase in physical activity, reduction in smoking and alcohol consumption, changes in sun protection behavior, total cholesterol, high-density lipoprotein (HDL) cholesterol, low-density lipoprotein cholesterol, non-HDL cholesterol, triglycerides, weight, BMI, body fat, lean mass, abdominal, waist and biceps circumference, abdominal skinfold thickness, tricipital skinfold thickness, dynamometry, sit to stand 5 repetitions test, perceived quality of life, acquired knowledge, self-efficacy, and usability.

For participants with T1D, hypoglycemic events (number of episodes of at least 15-minute duration with a glucose concentration of <70 mg/dL according to the CGM and manually registered episodes), HbA_1c_, average glucose (average glucose in mg/dL over the last 14 days), glucose management indicator (a measure of glycemic control calculated from average glucose, which is an estimation of HbA_1c_) and time in target range (% of time in the last 14 days that glucose is within (70-180mg/dL), below (<70 mg/dL), above (181-240 mg/dL), or significantly above (>240 mg/dL) the recommended target will be measured.

#### Direct Adherence

Adherence is measured by frequency of app use (number of times participants log in per day), duration of use (number of hours per day), and consistency of data recording (interactions with the app, like using the diary, changes in the questionnaire answers, and entering hypoglycemia data).

#### Behavior Change

Changes in dietary habits, tobacco and alcohol consumption, and sunscreen use are measured by diary entries. Changes in physical activity (steps) are automatically measured by the physical activity monitor.

#### Biomedical Outcomes

A blood test and physical examination were only carried out in Spain. A blood test was performed at the beginning and end of the study to measure blood count, creatinine, urea, glucose, glutamic pyruvic transaminase, glutamic oxaloacetic transaminase, gamma-glutamyl transferase, total cholesterol, HDL cholesterol, and triglycerides in all participants. Automated, routine colorimetric methods are used. Non-HDL cholesterol is calculated by subtracting HDL from total cholesterol, and low-density lipoprotein cholesterol is estimated using Friedewald’s formula [39]. HbA_1c_ was measured by High-Performance Liquid Chromatography (using Diabetes Control and Complications Trial standards, following National Glycohaemoglobin Standardisation Programme) in people with T1D only.

As part of the physical examination, the patient’s weight and height were measured to calculate BMI. Measurements of the abdomen, arm, and calf circumference are taken with a non-extensible measuring tape. Grip strength is assessed using a Jamar dynamometer, and a Holtain plicometer (Holtain Ltd) is used to measure the triceps skinfold. Body fat and muscle mass are estimated using a Tanita DC-360 (Tanita) bioimpedance meter. Arm muscle circumference is calculated from the triceps skinfold and arm circumference measurement. Muscle quality is calculated based on the muscle mass from the bioimpedance analysis and the grip strength measurement [40]. Finally, the functional test sit-to-stand with 5 repetitions is performed [41].

For participants with T1D, time in relevant ranges (see above), average glucose, glucose management indicator, and hypoglycemic events are recorded by the app from a CGM FSL 2 or 3 (Abbott). Hypoglycemic events are also recorded or confirmed manually.

#### Perceived Quality of Life

The EQ-5D questionnaire [42] is used to assess health-related quality of life in all participants. It consists of 2 parts: The first part contains 5 health dimensions (mobility, self-care, activities of daily living, pain and discomfort, and anxiety and depression). For each dimension of the EQ-5D, severity levels are coded: 1 if the answer choice is “no (I have) problems,” 2 if the answer choice is “some or moderate problems,” and 3 if the answer choice is “many problems.” The higher the score, the worse the health dimension. The second part of the EQ-5D is a scale from 0 to 100. On this scale, the individual should mark the point that best reflects their assessment of their overall health status, with 0 being the lowest assessment of their health status and 100 being the best.

The Life with Type 1 Diabetes (Vida con Diabetes tipo 1; ViDa1) [43], a questionnaire for health-related quality of life in patients with T1D, is also used for the group of individuals with T1D. The ViDa1 has 34 items that are grouped into 4 different dimensions: interference with life, self-care, well-being, and worry about the disease. It is a questionnaire that can be self-administered with a Likert-type response format in which a total score per subscale is obtained. Interference with life: (items 1-12), self-care (13-23), well-being (24-29), and worry about illness (30-34). Each item is scored from 1 “strongly disagree” to 5 “strongly agree.” For correction, the scores obtained in each subscale are added together. To ensure an accurate interpretation, the scoring for items 12, 23, and 27 must be reversed (a score of 1 indicates strongly agree, while a score of 5 indicates strongly disagree).

#### Knowledge and Attitudes

To measure diabetes self-management behaviors, the Summary of Diabetes Self-Care Activities (SDSCA) is used [44]. It consists of 11 items that address different areas of self-care present in people with diabetes mellitus, such as diet, physical activity, medication, self-testing of capillary glycemia, and smoking. It presents a response scale from 0 to 7, depending on the number of days that the person has carried out a certain behavior in the previous week. The smoking item has a dichotomous response. The questionnaire has no cutoff point, so each item must be assessed individually.

To assess the knowledge acquired about healthy habits, the European Health Literacy Survey is used [45]. The questionnaire consists of 16 questions that classify the degree of difficulty perceived by the participant in each task or situation as: 1 “very easy,” 2 “easy,” 3 “difficult,” 4 “very difficult,” or 5 “don’t know/no answer.”

#### Satisfaction and Feedback

The System Usability scale (SUS) [46] and mHealth App Usability Questionnaire (MAUQ) [47] are used. The SUS scale is a 10-item questionnaire scored on a 5-point Likert-type scale from 1 (strongly disagree) to 5 (strongly agree). The overall score ranges from 0 to 100. A system with a score above 85 is considered to have excellent usability. The MAUQ assesses the ease of use, interface, satisfaction with, and usefulness of mHealth apps for end users. It consists of 18 items, with 7 response options for each item, ranging from 1 “strongly disagree” to 7 “strongly agree.”

Table 1 summarizes the procedures associated with the trial.

**Table 1:** Study schedule.

Months and procedures	Screening	January (Initial visit app download)	February (Initial visit app download)	March	April	May (Final visit)	June (Final visit)
Informed consent	✓	—^a^	—	—	—	—	—
Demographics	✓	—	—	—	—	—	—
Medical history	✓	—	—	—	—	—	—
Physical examination^b^	—	✓	✓	—	—	✓	✓
Blood tests^b^	—	✓	✓	—	—	✓	✓
Body composition^b^	—	✓	✓	—	—	✓	✓
Questionnaires	—	✓	✓	—	—	✓	✓
Usability, engagement, and digital health literacy scales	—	—	—	—	—	✓	✓
Likert scale (primary outcome)	—	—	—	—	—	✓	✓
Data collection (glucose sensor and physical activity device)	—	✓	✓	✓	✓	✓	✓

^a^Not applicable.

^b^Procedures performed only in the Spain center.

### Statistical Analysis

Descriptive results will be presented as mean (SDs), or median (IQRs) for continuous variables, and frequencies and percentages for categorical variables. The primary endpoint is the difference in Likert score between the 2 groups in the self-reported achievement of the individual, self-defined aim at the end of the study. The intervention and control groups will be compared using a Student *t* test (2-tailed) or a Mann-Whitney *U* test. We will also perform linear regression to compare the groups with adjustment for the stratification factor center. For comparison of changes in variables measured at baseline and end of study, we will use linear regression or logistic regression adjusting for the baseline measurement [48]. Logarithmic transformation may be used. A *P* value below .05 will be considered significant.

An intention-to-treat analysis will be performed (including all randomized participants, regardless of their adherence to the intervention).

### Power and Sample Size

The sample size was calculated for the primary outcome (Likert scale from 1 to 10 with a target defined by the participant). A population of people with T1D was used as the reference population, based on a previous study [38,49]. The common SD in the intervention and control groups is assumed to be 2.07 [38]. With a significance level of 0.05 and a power of 0.8 in a 2-sided 2-sample *t* test (2-tailed), 47 subjects are necessary in both groups to detect a statistically significant difference greater than or equal to 1.3 units. Allowing for a potential loss to follow-up of 10%, we estimated a total required sample size of 52 participants in each group. Participants with T1D are only recruited in Spain. All 3 centers aimed to recruit an equal number of participants, that is, 36 participants each (aiming at a total of 108).

The analyses will primarily be performed on the total sample for common outcomes. Regarding the group of participants with T1D, as the study lacks the power to detect clinically relevant changes in T1D-specific clinical outcomes, no definitive conclusions will be drawn from these results. Instead, the results will inform sample size estimation and the design of a subsequent trial targeting the T1D population specifically.

### Ethical Considerations

It was submitted to and approved by the Las Palmas Ethics Committee in September 2024 (reference 2024-330-1). It was submitted to clinicaltrials.gov in June 2024 and approved with ID NCT06918444. An insurance policy was signed for the entire duration of the study, which was conducted during the first half of 2025.

Potentially eligible participants were given an informed consent form and an information sheet about the study, in the relevant language (Spanish, Norwegian, Romanian, or English). Those who agreed to participate and signed the form were enrolled in the study. Participants could withdraw from the study at any time. The study participants’ data were anonymized during the study. Participants were not paid for entering or remaining in the study.

The WARIFA project places strong emphasis on data protection, algorithmic support, user autonomy, and transparency in AI‑driven recommendations. Personal and health‑related data are processed under explicit informed consent in accordance with General Data Protection Regulation, with safeguards such as pseudonymization, encryption, strict access control, and privacy‑by‑design measures embedded throughout the system architecture. AI algorithms operate on validated, pseudonymized datasets within a secure environment, providing personalized risk assessments and lifestyle recommendations without engaging in diagnostic or therapeutic decision‑making, ensuring that users remain in control of their health choices and can withdraw consent or correct their data at any time. Transparency is supported through clear privacy policies, detailed consent procedures, and user‑facing explanations of data flows, processing purposes, and the nonclinical nature of the AI outputs, thereby promoting informed engagement and preserving user autonomy while maintaining high ethical and regulatory compliance standards.

## Results

**Figure 3:**
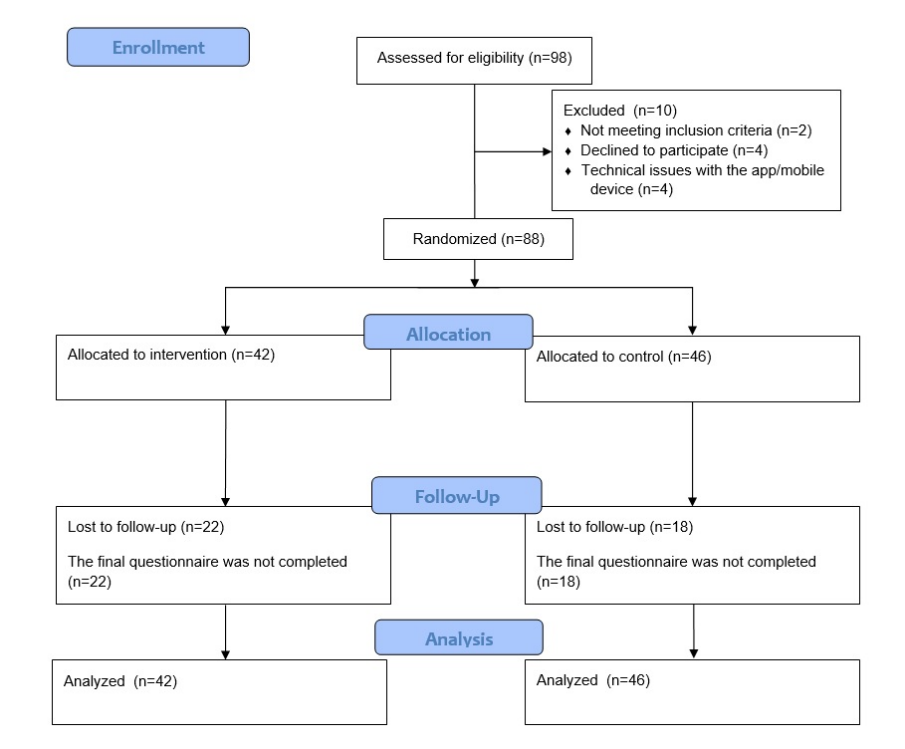
Flowchart of participant recruitment.

The clinical trial began in January 2025. A total of 88 participants were recruited from 3 centers: 34 from Spain, 34 from Norway, and 20 from Romania (Figure 3). Participants were randomized at a ratio of 1:1 to either the intervention or control group (42 participants in the intervention group and 46 in the control group). Each participant was observed for up to 12 weeks in total, with the follow-up period ending in June 2025. The data are currently being analyzed and are expected to be published in 2026.

## Discussion

### Anticipated Findings

The RCT we describe aims to assess the short-term effectiveness of the WARIFA app in promoting users’ healthy behaviors and self-management of T1D. Our hypothesis is that dynamic, personalized recommendations can achieve self-defined objectives and healthy behavior changes to a larger extent than general recommendations for all users.

Other mHealth apps have been used for behavior change and disease management in patients with hypertension and obesity [50,51], although they do not provide estimates of the risk of NCDs.

The main strengths of this trial are that it is conducted in 3 European countries and includes a fairly large and heterogeneous sample size, which should support the applicability of the results. Furthermore, the use of a low-intervention app and a physical activity monitor as control, as well as the double-blind nature of the trial, reduces the risk of bias. The WARIFA app can provide personalized risk estimations for the major NCDs over the next 10 years, as well as identifying the habits to change and the most effective way to do so for each individual. This is in contrast to the generic messages typically used in community-based prevention. The app is able to deliver these personalized messages to participants’ devices to encourage adherence. A key strength of the WARIFA project is its rigorous commitment to data protection, robust algorithmic support, user autonomy, and full transparency in AI‑driven recommendations.

We also acknowledge some limitations in the design of the trial. Although we believe that the number of participants is appropriate for an initial evaluation of the WARIFA app, it may not be sufficient to assess many of the secondary outcomes addressed, especially in T1D. Another limitation is that the biomedical outcomes (anthropometry and blood tests) are performed at one of the centers only, and these data will not be available for all participants. Restrictions also include the fact that the WARIFA app is only compatible with Android mobile phones, as well as certain activity devices and glucose sensors. mHealth apps have boomed in recent years as a tool that is free, voluntary, and easy to obtain. However, many users uninstall the app shortly after installation [52].

Many mHealth apps have demonstrated their effectiveness in promoting healthy habits and managing chronic diseases such as diabetes and cardiovascular conditions; most of them rely on short-term interventions and have limitations in terms of long-term applicability and accessibility for the entire population [53-56]. In contrast, the WARIFA app has been designed using an integrative, personalized approach that targets adults of all ages, including those with low digital literacy skills and seldom heard populations [57,58], areas that are often missed by similar apps. However, long-term applicability is not assessed.

Furthermore, the WARIFA app was developed by actively incorporating feedback from potential users and health care professionals through focus groups. This participatory process allowed the design, functionality, and content to be tailored to users’ real needs. This participatory process improves usability, acceptability, and scalability. WARIFA aims to promote healthy behaviors through a more inclusive and user-centered tool than many existing apps.

### Conclusions

This randomized, double blind, controlled, parallel-group trial assesses the short-term effectiveness of the WARIFA health app in promoting healthy behaviors. It will also allow us to identify its weaknesses and limitations, to further improve and develop this, or similar apps. A longer-term study would be needed to assess its long-term effects, including those on the risk for major NCDs, as well as its integration into health care systems.

## Abbreviations

AI: artificial intelligence
CGM: continuous glucose monitoring
COPD: chronic obstructive pulmonary disease
CVD: cardiovascular disease
EU: European Union
FSL: Free Style Libre
HbA1c: glycosylated hemoglobin A1c
HDL: high-density lipoprotein
MAUQ: mHealth App Usability Questionnaire
mHealth: mobile health
NCD: noncommunicable disease
RCT: randomized controlled trial
SDSCA: Summary of Diabetes Self-Care Activities
SPIRIT: Standard Protocol Items: Recommendations for Intervention Trials
SPIRIT-AI: Standard Protocol Items: Recommendations for Intervention Trials–Artificial Intelligence
SUS: System Usability Scale
T1D: type 1 diabetes
T2D: type 2 diabetes
ViDa1: Life with Type 1 Diabetes (Vida con Diabetes tipo 1)
WARIFA: Watching the Risk Factors

## Data Availability

The datasets generated or analyzed during this study are not publicly available due to privacy and data protection regulations.
